# Convergent cardiorespiratory neurons represent a significant portion of cardiac and respiratory neurons in the vagal ganglia

**DOI:** 10.3389/fcvm.2022.959815

**Published:** 2022-10-05

**Authors:** Asokan Devarajan, Ke Wang, Kassandra Shannon, Yujuan Su, Jamie Verheyden, Xin Sun, Marmar Vaseghi

**Affiliations:** ^1^UCLA Cardiac Arrhythmia Center, UCLA Health System, Los Angeles, CA, United States; ^2^Neurocardiology Research Center of Excellence, University of California, Los Angeles, Los Angeles, CA, United States; ^3^Department of Pediatrics, University of California, San Diego, La Jolla, CA, United States

**Keywords:** autonomic nervous, vagal, neurocardiology, cardiac neurons, respiratory neurons, cardiorespiratory neurons, convergent neurons

## Abstract

Significant cardiorespiratory coordination is required to maintain physiological function in health and disease. Sensory neuronal “cross-talk” between the heart and the lungs is required for synchronous regulation of normal cardiopulmonary function and is most likely mediated by the convergence of sensory neural pathways present in the autonomic ganglia. Using neurotracer approaches with appropriate negative control experiments in a mouse model, presence of cardiorespiratory neurons in the vagal (nodose) ganglia are demonstrated. Furthermore, we found that convergent neurons represent nearly 50% of all cardiac neurons and approximately 35% of all respiratory neurons. The current findings demonstrate a pre-existing neuronal substrate linking cardiorespiratory neurotransmission in the vagal ganglia, and a potentially important link for cardiopulmonary cross-sensitization, which may play an important role in the observed manifestations of cardiopulmonary diseases.

## Introduction

The autonomic nervous system innervates all visceral organs, regulating every aspect of their function and allowing them to operate in a coordinated fashion (1). A significant highway for afferent and efferent communication between the brain and the visceral organs is the vagus nerve, which carries cardiovascular and respiratory signals to the brainstem *via* sensory neurons in the vagal ganglia (nodojugular-complex, NJG). Mechanical interactions between the heart and lungs are well-established. Pathological processes, such as myocardial injury that affect the heart cause autonomic remodeling in the peripheral and central nervous system, leading to sympathetic activation as well as parasympathetic dysfunction (1). This autonomic remodeling results in progression of heart failure and arrhythmias (1–3). On the other hand, injury to the central nervous system, such as subarachnoid hemorrhage, can affect vagal neuronal density, leading to cardiac electrophysiological abnormalities However, while axonal branching is known to be ubiquitous in the brain (4), and specific cardiopulmonary preganglionic parasympathetic neurons that coordinate cardiopulmonary function have been identified in the nucleus ambiguous of the brainstem (5), little attention has been given to potential cardiac and respiratory interactions that may exist *via* a shared peripheral autonomic network. Using retrograde fluorescent labeling techniques, Tomney and colleagues reported evidence of branching post-ganglionic sympathetic efferent cardiopulmonary neurons in the canine sympathetic ganglia (6). Given the tight physiological coordination of the cardiorespiratory system and sensory innervation of both organs *via* the vagal nerve, we hypothesized that a significant portion of cardiac and respiratory neurons in the vagal ganglia may also be convergent, allowing for the processing of afferent inputs from both organs, and tested this hypothesis using several retrograde labeling techniques.

## Materials and methods

All animal experimental procedures were approved by the University of California at Los Angeles. C57BL/6J (000664) mice were purchased from the Jackson Laboratory. Benzyl ether, dichloromethane, and dispase II were purchased from Millipore Sigma. CTB-Alexa Fluor-555 was purchased from Thermofisher Scientific (Waltham, MA, USA). Collagenase I was obtained from Life Technology Corporation (Carlsbad, CA, USA). RetroAAV2-Td-tomato was procured from Boston Children’s Hospital Core (Boston, MA, USA). Retro-AAV9-GFP was from Addgene, (Watertown, MA, USA). Fast blue (FB) was obtained from Polyscience, (Warrington, PA, USA).

### Administration of neurotracers in the lungs and heart

Three months old mice were anesthetized with ketamine (100 mg/kg) and xylazine (10 mg/kg IP). Anesthetized mice were positioned on the surgical platform. A laryngoscope was used for direct visualization of the glottis. The trachea was intubated with a 20-gauge blunt catheter. For non-viral neuro-tracer approaches, mice (*n* = 6) received 30 μL of 2% FB intratracheally, following confirmation of intratracheal catheter placement *via* visualization of condensation on a hand-held mirror. After 11 days, cardiac injections of cholera toxin B Alexa Fluor-555 (CTB, *n* = 6) were performed in FB administrated mice, as previously described (7). Briefly, mice received carprofen (5 mg kg^–1^, s.c.) and buprenorphine (0.05 mg/kg, s.c.) 1 hour before surgery. Animals were anesthetized with isoflurane (induction at 5%, maintenance at 1–3%, inhalation), intubated, and mechanically ventilated. Core body temperature was maintained at 37^°^C. The surgical incision site was cleaned using 10% povidone-iodine and 70% ethanol. A left lateral thoracotomy was performed at the fourth intercostal space and the heart was exposed, cardiac injections of the left and right ventricles were performed with 10 μL of 0.1% CTB. The surgical wounds were closed with 6–0 sutures. Buprenorphine (0.05 mg/kg, s.c.) was administered daily for 2 days after surgery. Animals were sacrificed 3 days later and bilateral NJG were dissected and collected for imaging and fluorescence-activated cell sorting. For viral neurotracer studies, retro-AAV2-td-tomato (1 × 10^12 vg/mL, 1 in 5 dilutions) (*n* = 5) was administered intratracheally followed by cardiac retro-AAV9-GFP (1 × 10^13 vg/mL1 in 10 dilutions) injections after 2 days (*n* = 5). Three weeks later, bilateral NJGs were isolated. In two other groups of mice (*n* = 3 per group), only cardiac injections of neurotracers (CTB in one group and retro-AAV2-td-tomato in the other group) were performed. In the mice receiving CTB, NJG, lungs, and lumbar dorsal root ganglia (DRG) were isolated after 3 days, while in the group receiving retro-AAV2-td-tomato only, those organs were isolated after 21 days for imaging.

### Tissue clearing

Tissue clearing was performed using the iDISCO protocol (7). Briefly, fixed lungs lobes were dehydrated with graded methanol [20, 40, 60, and 80% methanol in H_2_O (vol/vol) sequentially, each for 1 h at room temperature], washed twice with 100% methanol for 1 hour at room temperature, and chilled at 4^°^C. Lungs were then incubated in 66% dichloromethane/33% methanol overnight, washed twice in 100% methanol for 1 h at room temperature, and chilled to 4^°^C. Following these steps, tissue was bleached with 5% H_2_O_2_ in methanol (vol/vol) overnight at 4^°^C. After bleaching, the lungs were again rehydrated with graded methanol, in series, followed by washes with 0.01 M PBS, and then dehydrated again with graded methanol, in series and, incubated in 66% dichloromethane/33% methanol for 3 h at room temperature. Then, the lung tissue was washed using 100% dichloromethane and stored in benzyl ether for imaging.

### Confocal imaging

Whole NJG or cryo-sectioned NJG (7 μm slices) and lumbar DRGs were fixed in 4% PFA. NJGs, lumbar DRGs, and, cleared lung tissue were imaged using a confocal laser scanning microscope (Zeiss, LSM 880). Individual slices were analyzed from non-viral tracer (CTB and FB) and viral tracer (retro-AAV2-td-tomato and retro-AAV9-GFP) administrated mice using Z-stack imaging with confocal microscopy. For CTB imaging, excitation wavelength of 633 nm (filter wavelength range of 562–642 nm) and for FB imaging, excitation wavelength of 561 nm (laser DPSS 561-10, GFP filter wavelength range of 410–513 nm) was used. For GFP imaging, laser wavelength of 549 nm (filter 491–606 nm) and for td-tomato imaging, excitation wavelength of 561 (laser HENE633, filter range of 566–697 nm) was used. Images were processed with Zen 2 (Zeiss) software. Labeled neurons were manually quantified for 3 animals that received retrograde viral tracers and 5 animals that received non-viral (CTB/FB) tracers.

### Fluorescence-activated cell sorting using flow cytometry in CTB/FB labeled ganglia

NJG isolated from CTB/FB mice were digested with collagenase I, and Dispase II at 37°C for 45 min, washed with L-15 medium, gently triturated with glass aspiration pipettes of decreasing diameter, and filtered with a 40 μm cell strainer. The cell suspensions were sorted on a 5 Laser SORP Aria II (488, 562,633, 405, and 350 nm) 4-way 4^°^C chilled sorter. The 670/14 635LP filter on the 562 nm laser was used to detect CTB-555 and the 405 nm laser with the 450/50 filter set was used for FB detection.

### Statistical analysis

All values are expressed as mean ± standard mean error (SEM). One-way ANOVA with Tukey multiple comparison test was used for multiple group comparisons. PRISM (GraphPad) was used for all statistical analyses.

## Results and discussion

**FIGURE 1:**
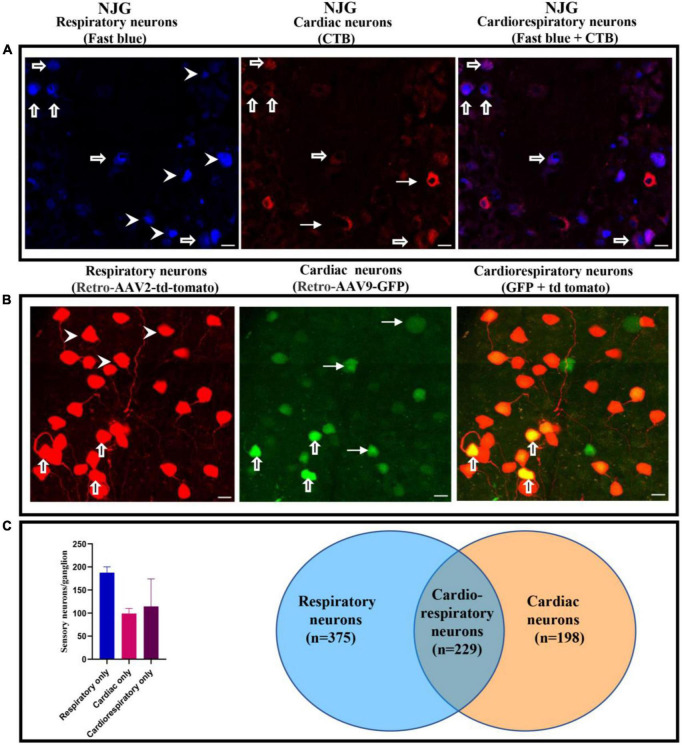
Presence of cardiorespiratory neurons in the NJG. **(A)** NJGs were isolated from FB (*n* = 6) and CTB administered mice (*n* = 6), cryosectioned (7 um slices), and imaged using confocal microscopy. Presence of respiratory, cardiac, and cardiorespiratory neurons (dual labeling of CTB and FB) was observed in all ganglia (Bars = 20 μm). Thick arrows point to cardiorespiratory neurons, arrowheads to respiratory only, and thin arrows to cardiac only neurons. **(B)** NJGs were isolated from retro-AAV2-td-tomato (*n* = 5) and retro-AAV9-GFP (*n* = 5) mice. Individual slices were imaged by confocal microscopy. Retro-AAV2-td-tomato and retro-AAV9-GFP also demonstrated evidence of neurons with dual labeling (Bars = 20 μm). Thick arrows point to examples of convergent cardiorespiratory neurons, arrowheads to examples of respiratory only, and thin arrows to examples of cardiac only neurons. **(C)** Flow cytometry data from NJG (2 separate groups of 3–4 mice) also confirmed evidence of convergent neurons.

Cardiac and respiratory neuronal labeling was observed with both CTB/FB and retro-AAV tracers using confocal imaging (Figures 1A,B and Supplementary Figure 1). Notably, colocalization of CTB and FB and colocalization of retro-AAV9-GFP and retro-AAV2-td-tomato tracers were observed in multiple NJG neurons in all animals undergoing both cardiac and respiratory tracer administration, demonstrating the existence of convergent neurons. Image quantification of neurons showed that that mice that received a non-viral neuronal tracer (CTB/FB) exhibited two to threefold higher neuronal labeling as compared to mice that received a retro-AAV tracers (Table 1). This is likely due to the different mechanisms of retrograde transport employed by these tracers. Non-viral neurotracers, such as CTB and FB, directly bind with host biomolecules. CTB binds with GM1 gangliosides that are present on the surface of the plasma membranes, whereas FB binds to nucleic acids. In the case of retrograde viral neurotracers (retro-AAVs), at least three physiological barriers must be crossed by the AAV to transduce host cells: (1) AAV has to bind with the plasma membrane, (2) AAV internalization has to occur *via* a dynamin-dependent mechanism (for AAV2 it has to be the endocytosed *via* clathrin-coated vehicles), and (3) the virus has to enter into the nucleoplasm through the nuclear pore and then integrate its genome into the nucleic acid (8). Finally, the AAV virus that has successfully integrated expresses the fluorescent protein allowing for detection (8). Therefore, it is not surprising that retrograde viral tracers have lower expression/labeling than non-viral tracers. Regardless of the type of neurotracer administered, the number of labeled respiratory neurons was approximately twofold higher than the number of cardiac or cardiorespiratory neurons. This is likely due to the wider distribution of FB throughout the lungs following intratracheal administration. With both viral and non-viral neurotracer techniques, cardiorespiratory neurons represented a significant portion of cardiac and respiratory neurons in the NJG complex. The presence of convergent-dual-labeled CTB/FB neurons was also confirmed by flow cytometry. Flow cytometry detected 185 ± 17 labeled respiratory neurons, 99 ± 11 labeled cardiac neurons and 114 ± 59 dual-labeled cardiorespiratory neurons present in per nodose ganglion. Similar to results obtained from imaging techniques, the number of respiratory neurons was 1.8 times greater than cardiac neurons on flow cytometry analysis (Figure 1C, raw data in Supplementary Figure 1).

**TABLE 1:** Quantification of cardiac, respiratory, and dual labeled cardiorespiratory neurons on confocal imaging in the mouse NJG complex.

Non-viral neurotracers		
Respiratory neurons/Ganglion	Cardiac neurons/Ganglion	Cardiorespiratory neurons/Ganglion
112 ± 19	55.3 ± 8*	30 ± 7***

**Viral neurotracers**		

**Respiratory neurons/Ganglion**	**Cardiac neurons/Ganglion**	**Cardiorespiratory neurons/Ganglion**

34 ± 3	19 ± 3***	14 ± 2***

CTB/FB group: *n* = 5 animals; retro-AAV2-td-tomato and retro-AAV9-GFP group: *n* = 5 animals; *P < 0.05 for comparison of cardiac to respiratory neurons using non-viral techniques, ***P < 0.001 for comparison of cardiorespiratory neurons to respiratory neurons. The number of cardiorespiratory compared to number of cardiac neurons was not statistically different.

**FIGURE 2:**
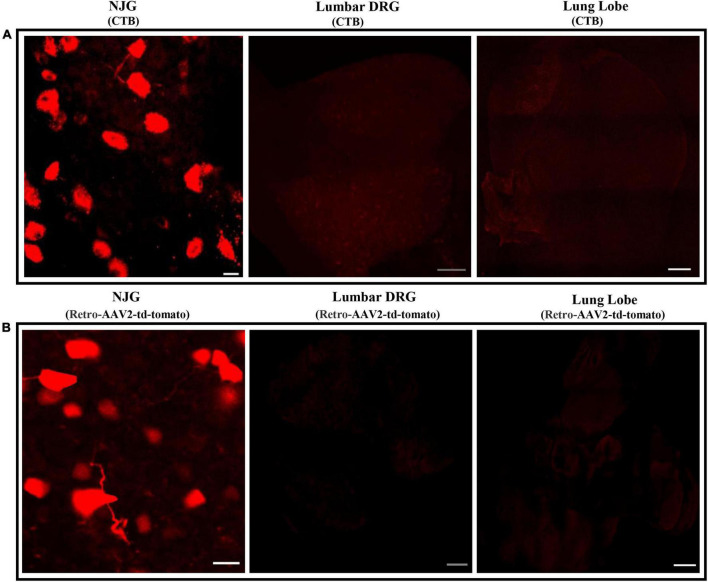
Absence of neuronal labeling in lumbar DRGs and the lungs following cardiac injections of neurotracers. **(A,B)** The lungs, lumbar DRGs, and NJG were isolated from mice after CTB (*n* = 3) or cardiac retro-AAV2-td-tomato (*n* = 3) injections and maximum intensity projections were imaged. While neuronal labeling in the NJGs was clearly observed, no labeling of neurons or nerves was seen in the lungs (confirming lack of leakage of dye) or in the lumbar DRGs (confirming lack of significant leakage into the systemic circulation). NJG: Bars = 20 μm. Lumbar DRGs: Bars = 100 μm. Cleared lung tissue: Bars = 500 μm.

To ensure that the labeling of convergent neurons was not due to leakage of dye from the heart into the lungs or systemic circulation, analysis of the NJGs, lumbar DRGs (ganglia that are not associated with cardiac innervation) and cleared lung tissue was performed in mice injected with only cardiac CTB and retro-AAV. The results revealed that in all mice, labeled NJG neurons were observed, while no labeling (Figures 2A,B) of nerves or neurons was seen in the cleared lung tissue or the lumbar DRGs, suggesting that localized or systemic spread of CTB/AAV was unlikely to explain convergent neuronal labeling. Our results demonstrate that convergent cardiorespiratory neurons represent a significant portion of all cardiac and respiratory sensory neurons in the peripheral vagal ganglia, a novel finding of this study. A recent study suggested that neurovisceral organs may have parallel processing within peripheral ganglia, with neurotransmission convergence occurring primarily at the level of the brainstem (9). Given the significant portion of convergent cardiorespiratory neurons noted in this study, it’s possible that an important degree of cardiorespiratory coordination may occur at the vagal ganglia and other shared peripheral ganglia. These neurons appear to be a specific subset of sensory neurons that innervate both organs and may play an important role in synchronizing cardiorespiratory function.

It is established that pulmonary pathologies affect cardiac function, and conversely, cardiac disease is known to negatively impact respiratory processes. Until now, much focus has relied on mechanical interactions between these organs. Yet, many patients demonstrate evidence of multi-organ autonomic dysfunction after single organ pathology, which could be mediated *via* their shared autonomic sensory neuronal network. For example, patients with chronic obstructive pulmonary disease demonstrate abnormal heart rate recovery after exercise (10). The prevalence of cardiac arrhythmias, especially atrial fibrillation, where vagal neurotransmission plays an important role, is increased in these patients (11). Conversely, Cheyne-Stroke respiration and sleep apnea are associated with the occurrence of atrial fibrillation and increased mortality in the setting of heart failure (12). Most mechanistic studies, however, have focused on the effects of a single organ pathology on the autonomic nervous system and vice-versa. The specific effects of cardiac pathology on the autonomic nervous system, for example, have been extensively studied. Myocardial injury is known to cause remodeling of the cardiac autonomic nervous system, leading sympathetic activation and parasympathetic dysfunction (1). In a chronic porcine infarct model, myocardial infarction was associated with significant functional and structural remodeling of nodose ganglia neurons that transmit cardiac nociceptive signals, reducing cardiac nociceptive neurotransmission (13). Nodose ganglion degeneration after subarachnoid hemorrhage is associated with coronary vasospasm, and ischemic neurodegeneration of the vagal ganglia can lead to ventricular arrhythmias during subarachnoid hemorrhage (14, 15). While these studies have clearly demonstrated a two-way relationship between the heart and the nervous system *via* the peripheral vagal ganglia, data regarding multi-organ interactions through the peripheral autonomic nervous system are sparse. A possible mechanism for dual labeling of neurons may be due to branching axons, that has also been observed in the sympathetic chain of canines and is known to exist in the brain (6, 16).

Our study suggests that many of the cardiac and respiratory sensory innervation share the same neurons at the level of the peripheral vagal ganglia, and processes that affect the heart or the lung may, in fact, affect other organs through this peripheral neural link. The current study’s findings open up a potentially new and important avenue of investigation in the context of cardiopulmonary disease, linking cardiorespiratory interactions *via* their shared sensory neurons in the NJGs. Several studies are underway in our laboratory to generate molecular maps of cardiorespiratory neurons in health and disease. Identifying neural pathways and functions of these neurons may shed important light on how these organs are coordinated through the autonomic network in health and disease.

## Data availability statement

The original contributions presented in this study are included in the article/Supplementary material, further inquiries can be directed to the corresponding author.

## Ethics statement

The animal study was reviewed and approved by the University of California at Los Angeles Animal Research Committee.

## Author contributions

AD, KW, and KS performed the experiments and analyzed data, and drafted manuscript. YS and JV assisted with protocols creation and experiments, and edited the manuscript. XS assisted with design of studies, data interpretation, and editing of the manuscript. MV oversaw design of the study, protocols, experiments, data interpretation, and drafted and finalized the manuscript. All authors contributed to the article and approved the submitted version.
