# Supplementary material for: Convergent cardiorespiratory neurons represent a significant portion of cardiac and respiratory neurons in the vagal ganglia
Source: Front Cardiovasc Med. 2022 Oct 5;9:959815. doi: 10.3389/fcvm.2022.959815 (PMC9579688; doi:10.3389/fcvm.2022.959815)
Supplement: Supplementary file 1 [file Image_1.pdf]

Supplemental Figures

Supplemental Figure 1.

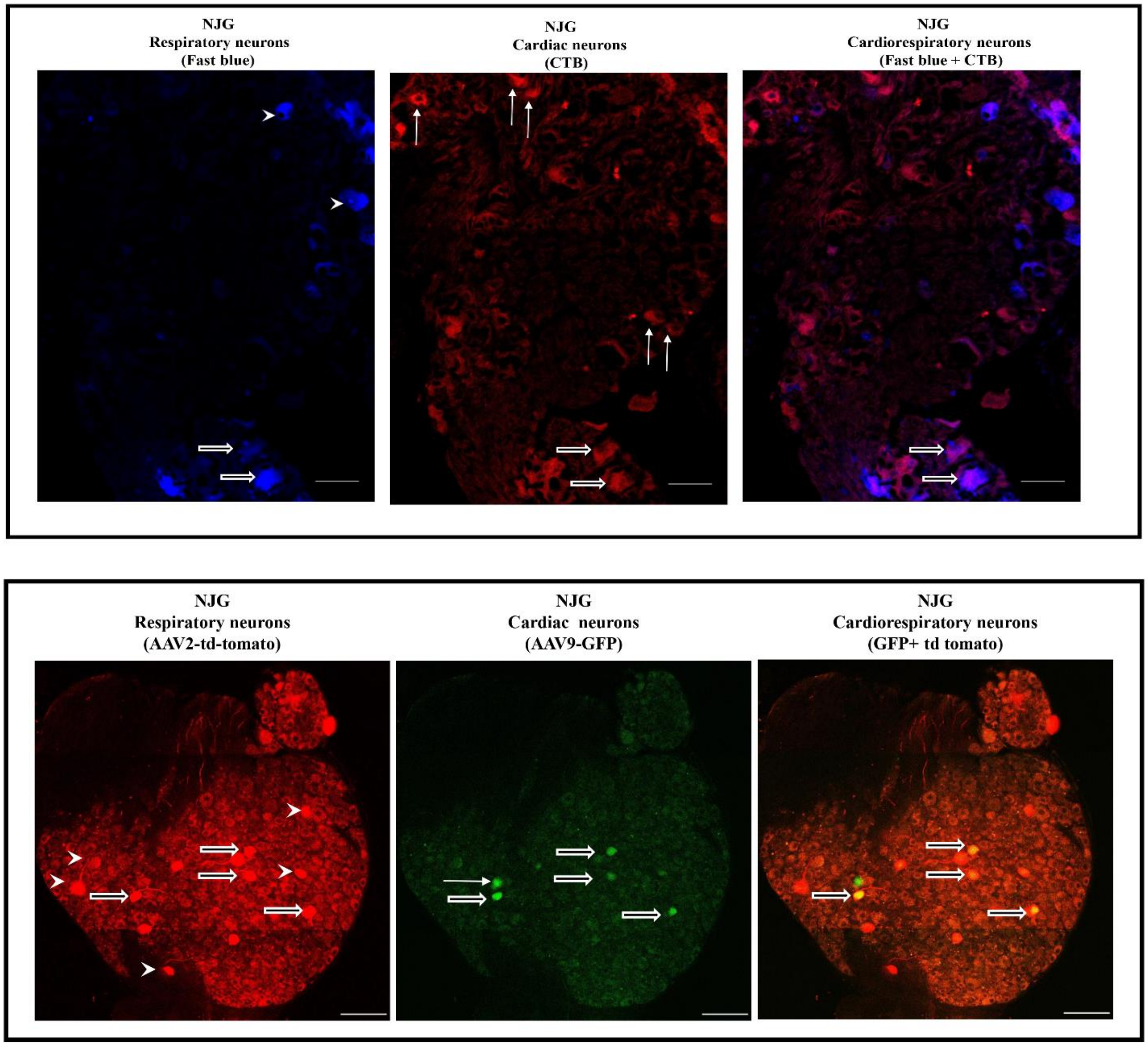

Supplemental Figure 1. Top panel: confocal imaging of nodose ganglia from a mouse (different animal than figure 1) that underwent both cardiac CTB injections and intratracheal administration of fast blue demonstrate labelling of respiratory neurons (arrowheads), cardiac neurons (thin arrows) and dually labelled cardiorespiratory neurons (thick arrows). **Bottom panel:** nodose ganglia from a mouse who underwent both intratracheal administration of retro-AAV2-td-tomato and cardiac injections of retro-AAV9-GFP show evidence of respiratory (arrow heads), cardiac (thin arrow) and dual-labelled cardiorespiratory neurons (thick arrows). Note that arrows point to a few examples of each type of labelled neurons.

Supplemental Figure 2.

Negative control sample

A

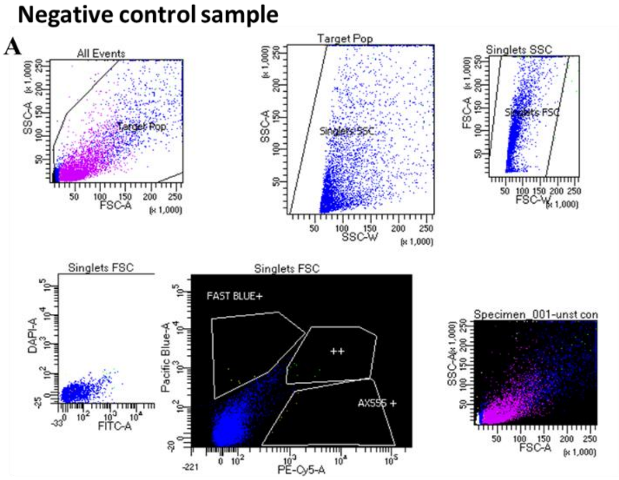

Tube: unst cont

Population	#Events	%Parent	%Total
All Events	10,773	####	100.0
Target Pop	7,429	69.0	69.0
Singlets SSC	7,426	100.0	68.9
Singlets FSC	7,414	99.8	68.8
P5	172	2.3	1.6
P6	0	0.0	0.0
P7	13	0.2	0.1
P8	577	7.8	5.4
FAST BLUE+	7	0.1	0.1
AX555 +	7	0.1	0.1
++	7	0.1	0.1
Q1	0	0.0	0.0
Q2	2	0.0	0.0
Q3	7,071	95.2	65.6
Q4	356	4.8	3.3
P4	4,623	42.9	42.9

Experimental sample

B

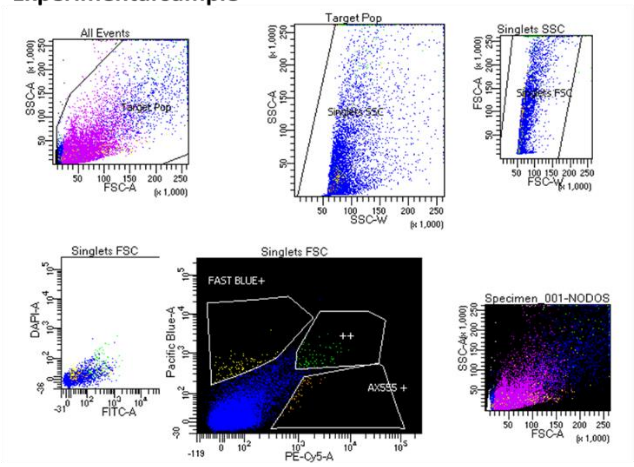

Tube: NODOSE CAR

Population	#Events	%Parent	%Total
All Events	55,601	####	100.0
Target Pop	31,870	57.3	57.3
Singlets SSC	31,865	100.0	57.3
Singlets FSC	31,859	100.0	57.3
P5	260	0.8	0.5
P6	0	0.0	0.0
P7	61	0.2	0.1
P8	1,507	4.7	2.7
FAST BLUE+	93	0.3	0.2
AX555 +	44	0.1	0.1
++	72	0.2	0.1
Q1	0	0.0	0.0
Q2	0	0.0	0.0
Q3	31,392	98.5	56.5
Q4	478	1.5	0.9
P4	23,576	42.4	42.4

C

Experiment : Murine Neuronal cells 04/06/20
Specimen : Specimen_001
Tube : NODOSE CAR
Sort Layout : Sort Layout_001
Application : FACSDiva Version 8.0.2

Sort Report

Report Date : 2020.04.06 at 15:44:34
Device : 4 Tube
User ID : Vaseghi
Cytometer : FACSAriaII (P69500098)

Sort Settings

Sort Setup	100 micron	Precision	HI purity
Frequency	32.6	Yield Mask	0
Amplitude	4.5	Purity Mask	16
Phase	0.00	Phase Mask	0
Drop Delay	27.54	Single Cell	Off
Attenuation	Off	Plates Voltage	4,321
Sweet Spot	Off	Voltage Centering	181
Breakoff	320	Sheath Pressure	23.00
Gap	12		

Side Stream Voltage (%)

Far Left	Left	Right	Far Right
54.00	23.00	23.00	50.00

Neighboring Drop Charge (%)

2nd	3rd	4th
16.00	6.00	3.00

Acquisition Counters

Threshold Count	677412
Processed Events Count(evt)	115460
Electronic Aborts Count(evt)	1265
Sort Elapsed Time(hh:mm:ss)	00:11:11

Sort Counters

	Far Left	Left	Right	Far Right
Sort Rate(evt/s)	0	0	0	NA
Conflicts Count(evt)	107	31	122	NA
Conflicts Rate(evt/s)	0	0	0	NA
Efficiency(%)	0	0	0	NA

Sort Layout

	Respiratory neuron	Cardiac neuron	Cardiorespiratory neuron	
	Far Left	Left	Right	Far Right
	FAST BLUE+ : 1202	AX555 + : 528	++ : 1049	

Supplemental Figure 2

(A) For Negative control (n=4), NJGs were isolated from mice that neither received CTB nFB and were digested with collagenase I, and Dispase II at 37°C for 45 min, washed with L-15 medium, gently triturated with glass aspiration pipettes of decreasing diameter, and filtered with a 40 μm cell strainer. The cell suspensions were sorted on the BD Influx system using the BD FACS Software. B) For experimental samples (n=4), NJGs were isolated from mice that received FB and CTB as described in the method section. Single-cell suspensions were prepared as described above. Cells were sorted on the BD Influx system using the BD FACS Software. C) Total number of cardiac neurons, respiratory neurons, and cardiorespiratory neurons were tabulated
